# Spontaneously Fluctuating Motor Cortex Excitability in Alternating Hemiplegia of Childhood: A Transcranial Magnetic Stimulation Study

**DOI:** 10.1371/journal.pone.0151667

**Published:** 2016-03-21

**Authors:** William M. Stern, Mahalekshmi Desikan, Damon Hoad, Fatima Jaffer, Gionata Strigaro, Josemir W. Sander, John C. Rothwell, Sanjay M. Sisodiya

**Affiliations:** 1 NIHR University College London Hospitals Biomedical Research Centre, Department of Clinical and Experimental Epilepsy, UCL Institute of Neurology, London, WC1N 3BG, United Kingdom; 2 Epilepsy Society, Chalfont St Peter, SL9 0RJ, United Kingdom; 3 Sobell Department of Motor Neuroscience and Movement Disorders, UCL Institute of Neurology, London, United Kingdom; 4 MRC Centre for Neuromuscular Diseases, UCL Institute of Neurology, London, WC1N 3BG, United Kingdom; 5 Department of Translational Medicine, Section of Neurology, University of Piemonte Orientale “A. Avogadro”, Novara, Italy; 6 Stichting Epilepsie Instellingen Nederland (SEIN), Heemstede, Netherlands; University of Toronto, CANADA

## Abstract

**Background:**

Alternating hemiplegia of childhood is a very rare and serious neurodevelopmental syndrome; its genetic basis has recently been established. Its characteristic features include typically-unprovoked episodes of hemiplegia and other transient or more persistent neurological abnormalities.

**Methods:**

We used transcranial magnetic stimulation to assess the effect of the condition on motor cortex neurophysiology both during and between attacks of hemiplegia. Nine people with alternating hemiplegia of childhood were recruited; eight were successfully tested using transcranial magnetic stimulation to study motor cortex excitability, using single and paired pulse paradigms. For comparison, data from ten people with epilepsy but not alternating hemiplegia, and ten healthy controls, were used.

**Results:**

One person with alternating hemiplegia tested during the onset of a hemiplegic attack showed progressively diminishing motor cortex excitability until no response could be evoked; a second person tested during a prolonged bilateral hemiplegic attack showed unusually low excitability. Three people tested between attacks showed asymptomatic variation in cortical excitability, not seen in controls. Paired pulse paradigms, which probe intracortical inhibitory and excitatory circuits, gave results similar to controls.

**Conclusions:**

We report symptomatic and asymptomatic fluctuations in motor cortex excitability in people with alternating hemiplegia of childhood, not seen in controls. We propose that such fluctuations underlie hemiplegic attacks, and speculate that the asymptomatic fluctuation we detected may be useful as a biomarker for disease activity.

## Introduction

Alternating hemiplegia of childhood (AHC) is a rare, severe neurodevelopmental disease with characteristic transient and unpredictable episodes of hemiplegia or tetraplegia. The designation is misleading; although it presents during childhood, affected children continue to manifest symptoms and signs of the condition into adulthood. The condition is also associated with other paroxysmal disturbances such as epileptic seizures, tonic or dystonic spells, abnormal eye movements, dyspnoea and transient autonomic dysfunction [1,2]. Symptoms typically improve following sleep. We have recently demonstrated dynamic alterations in the ECG, unrelated to hemiplegic episodes or epileptic seizures, suggesting paroxysmal electrophysiological dysfunction affecting heart as well as brain in AHC [3]. There is typically also permanent neurological dysfunction, including developmental delay, intellectual difficulties, and in some cases movement disorder (for instance choreoathetosis, dystonia or ataxia). Epileptic seizures occur in 18%-50% of those with AHC [1,2,4,5]. Disease prevalence is estimated at 1:1,000,000, which may be an underestimate caused by variability in clinical presentation, lack of awareness of the condition, and frequent misdiagnosis as epilepsy or cerebral palsy [6]. A recent Danish study suggested a prevalence of 1:100,000 [7].

Standard neuroimaging is typically normal in AHC [2,1,8,9], although abnormalities such as non-specific cerebral atrophy, vermian atrophy, hippocampal sclerosis and cortical dysplasia have been reported [9]. Single-photon emission computed tomography (SPECT) scanning shows ictal cerebral hypoperfusion [8,10].

Mutations in the *ATP1A3* gene, encoding Na+,K+-ATPase α3, account for around 80% of cases [5,11–14]; most are *de novo*. The encoded protein, the α3 subunit of Na^+^K^+^ATPase, is expressed widely in neuronal and cardiac tissue, contributing to trans-membrane sodium and potassium ion gradients, which are vital for cellular excitability [15].

The mutations causing AHC do not seem to affect gene expression, but instead modify protein function [5,16]. AHC-causing *ATP1A3* mutations cluster in particular gene regions, leading to speculation that a specific change in protein function causes the condition. Conversely, mutations causing rapid-onset dystonia parkinsonism, an allelic condition caused by *ATP1A3* mutations, do not cluster in particular gene regions. The specific mutation present may influence disease severity [12,14,16,17].

Various approaches have been employed to understand the effects of AHC-causing *ATP1A3* mutations on the function of the protein and on cells and model organisms. Cell-based assays show a reduction in ADP production [5], and a reduction in transmembrane current [18], both implying reduced activity of the Na+,K+ ATPase transport mechanism. Structural modelling of mutant proteins has also suggested that mutations causing AHC may lead to impaired Na+ and K+ transport [19]. In vitro and in silico modelling suggest that the most common AHC-causing mutations cause loss of forward cycling and dominant negativity, and that loss of proton transport may be correlated with disease severity [20].

Mutations in *ATP1A3* have been studied in animal models including mice, drosophila and zebrafish, and lead to varying degrees of neurological impairment including seizures and motor abnormalities. Some animal models exhibit a pathological response to stress, including seizures and balance problems (mice) and paralysis (drosophila) [16]. One mouse model with heterozygous mutation (*Atp1a3*^*Myk/+*^) demonstrates frontal hypometabolism, as well as reduced thalamocortical connectivity [21]. A novel knock-in mouse model carrying the most common heterozygous mutation causing AHC (p.D801N) was reported recently; this mouse displays many of the clinical features of AHC, and hippocampal slices show increased excitability in response to electrical stimulation [22].

Despite this progress, it is still not known how *ATP1A3* mutations cause the clinical phenotype of AHC and in particular the hemiplegic attacks. The clinical pattern of weakness, the resolution of symptoms during sleep, the other neurological manifestations of the condition, the expression of the causative gene and SPECT studies together point to a central mechanism for the hemiplegic attacks, with motor cortex a possible substrate.

Transcranial Magnetic Stimulation (TMS) can be used non-invasively to stimulate motor cortex, triggering a motor evoked potential (MEP) in a contralateral target muscle. The ease of activation of motor cortex is a measure of cortical excitability, while paired pulse paradigms test inhibitory and excitatory intracortical circuitry [23].

We used TMS to probe the mechanisms of hemiplegic attacks in people with AHC, and hypothesised that TMS would detect fluctuations in motor cortical excitability not seen in controls.

## Methods

The study was approved by National Research Ethics Service Committee London–Camden and Islington. TMS parameters were within the current safety guidelines [24]. Written informed consent was obtained. In people with mild learning difficulties, an approved adapted information sheet and consent form were used. In those lacking capacity, a relative was asked to provide assent. A safety questionnaire was completed for each participant.

Over a two year period, nine people with AHC were recruited from outpatient clinics, referrals and the Alternating Hemiplegia of Childhood UK support group. Eight were successfully tested, one was unable to cooperate. Ten healthy control subjects, on no medication, were recruited from a local database of volunteers. Ten consecutive people with epilepsy, on anti-epileptic medication, were recruited from clinic as further controls; none had a convulsive seizure in the 48 hours preceding or following testing. Intellectual difficulties were categorized as severe, moderate, mild, borderline or none according to ICD-10 guidelines [25]. Where formal neuropsychometry was unavailable, the degree of intellectual difficulties was assessed by a clinician blinded to the study outcome.

TMS was performed using two Magstim 200^2^ stimulators connected using a Bistim module, with a D70 alpha coil *(Magstim*, *Whitland*, *UK)*, controlled by Micro 1401 hardware and Signal software (*Cambridge Electronic Design*, *Cambridge*, *UK)*. EMG data were recorded from the contralateral adductor pollicis brevis (APB) muscle using surface electrodes, amplified using a Cambridge Electronic Design 1902 EMG amplifier, filtered using a 20Hz to 1kHz bandpass filter and 50Hz notch filter, and digitised at 2kHz for offline analysis.

Motor cortex was identified initially using skull landmarks. The motor hot-spot for the APB muscle, defined as the point on the scalp where stimulation produced the largest and most consistent EMG response, was identified. The hot-spot was marked on a tight-fitting cap to ensure a consistent site of stimulation. Where possible, the site was also marked using an ANT Visor neuronavigation system (ANT Neuro, Enschede, Netherlands); this included all controls and three out of five people with AHC who tolerated full testing. Data for each subject were collected during one sitting, in which the marked cap was not moved; only one subject was tested on two separate occasions, as outlined in the results section.

MEP was defined as the discrete EMG response seen in the target muscle 20-30ms after a TMS stimulus applied to the motor hot-spot. MEP amplitude was measured peak-to-peak and MEP latency was defined as the time interval between the TMS pulse and the first deviation of greater than three times the amplitude of the greatest fluctuation in the baseline resting EMG during 50ms prior to the stimulus.

These paradigms were used:

1. Resting Motor Threshold (rMT)
  With the subject relaxed, stimulation intensity was increased from 40% total machine output in 2% increments until a reliable EMG response was obtained. The intensity was then decreased in 1% increments until rMT was found, defined as the minimum TMS stimulus intensity required to give a peak-to-peak EMG amplitude of 0.1mV or greater in at least 5 out of 10 trials from the relaxed target muscle. rMT is a correlate of motor cortex excitability, with a low rMT corresponding to high excitability and vice versa [23].
2. Stimulus response curve
  EMG responses were measured with a range of TMS intensities (80%, 90%, 100%, 110%, 120% of rMT). Ten trials were performed at each intensity, in a randomised order. Average EMG amplitudes were plotted against stimulus size. This provides a more robust measure of neuronal excitability, using a wider range of stimulus intensities [23].
3. Short interval intracortical inhibition (SICI) and Intracortical Faciliation (ICF)
  A subthreshold conditioning stimulus (80% rMT) was followed by a suprathreshold test stimulus (110% rMT) with an inter-stimulus interval of 2ms (SICI) and 10ms (ICF). Single, unconditioned pulses were used as a control paradigm. Ten trials of each paradigm were performed and averaged (a total of 20 paired pulses and 10 single pulses). The conditioned EMG amplitude is normalised to the unconditioned EMG amplitude, and is typically reduced in SICI and increased in ICF. SICI is mediated by GABA_A_-ergic intracortical circuits [26], while ICF is mediated by glutamatergic intracortical circuits, along with a possible reduction in GABA-ergic inhibition [23].
4. Long interval intracortical inhibition (LICI)
  A supra-threshold conditioning pulse (110% rMT) was followed by a supra-threshold test pulse (110% rMT) with an inter-stimulus interval of 100ms; ten trials were performed. The EMG response to the test pulse was normalised to that generated by the conditioning stimulus; the conditioned response is typically reduced. This inhibition is mediated by GABA_B_-ergic intracortical circuits [23].

Stimulus-response curves were generated at two time points (Time 1 and Time 2). The stimulus-response curve for all was measured immediately after the motor threshold had been determined, and this was taken as Time 1. If a change in excitability was later detected, the stimulus-response curve was repeated and this was taken as Time 2. If no change was detected, the stimulus-response curve was repeated at the end of the testing session (an interval of 30 minutes), and this was taken as Time 2.

The mean total number of pulses administered to collect a full data-set in a single hemisphere was 318 (range 302–328). Subjects were required to sit still and remain alert with their eyes open, with the target muscle relaxed. Cooperation was confirmed by investigator observation and by EMG monitoring.

### Statistical analysis

Results for cases and controls were compared using unpaired t-tests (rMT, SICI, ICF, LICI).

Where one individual was tested at two time-points, these results were compared using paired t-tests (MEP size, MEP latency). For stimulus response curves, a multivariate ANOVA was used to adjust for multiple comparisons. P values of 0.05 or lower were interpreted as statistically significant. All analysis was performed using IBM SPSS Statistics (Version 22.0, Armonk, NY).

## Results

### Subjects

Nine adults with a clinical diagnosis of AHC (A1-A9) underwent TMS. Five tolerated TMS well enough for a complete dataset to be collected (two both hemispheres, three single hemisphere); one (A2) was initially tested using a clinical protocol, before also later undergoing TMS using our research protocol on a separate occasion. Three further people with AHC only tolerated testing of rMT, as they found testing uncomfortable and did not wish to continue. One person with more severe intellectual difficulties did not tolerate any interpretable testing. Eight of the nine had known *ATP1A3* mutations; one had a clinical diagnosis of AHC but no mutation was identified with whole genome sequencing (see Table 1). Ten consecutive people with treated epilepsy but not AHC (C1-C10) and ten healthy subjects on no medication (C11-20) were recruited as controls.

**Table 1:** Age, sex, mutation (subjects with AHC only), epilepsy diagnosis (epilepsy control subjects only), TMS paradigms tested, medication at time of testing and degree of intellectual difficulties for all subjects.

**Subjects with AHC**					
Subject number	Sex, Age	*ATP1A3* mutation	Testing Performed	Medication at time of testing (mg/day)	Intellectual Difficulties
A1	M, 18	c.2401G>A	rMT	None	Mild*
A2	F, 27	c.2431T>C	rMT, SRC, SICI, ICF, LICI	TPM 150, PHT 125, BAC 30, FLN 15	Mild*
A3	F, 31	None detected	rMT, SRC, SICI, ICF, LICI	ZNS 150, FLN 40, PZT 1, BAC 100,	None
A4	F, 36	c.2839G>A	rMT, SRC, SICI, ICF, LICI	BAC 10	Mild
A5	M, 19	c.2314A>C	rMT	TPM 75, FLN 10, PZT 1, OMP 20	Mild
A6	F, 35	c.2318A>G	rMT, SRC, SICI, ICF, LICI	FLN 7.5	Mild
A7	F, 18	c.2839G>A	rMT	None	Mild*
A8	M, 18	c.1072G>A	Testing impossible	PHT 300, VPA 1400	Severe*
A9	F, 33	c.1010T>G	rMT, SRC, SICI, ICF, LICI	LTG 400, PGB 200, AZM 200	None
**Control subjects with epilepsy**					
Subject number	Sex, Age	Epilepsy Diagnosis	Testing Performed	Medication at time of testing (mg/day)	Intellectual Difficulties
C1	M, 18	Focal Symptomatic	rMT, SRC, SICI, ICF, LICI	VPA 1000, PGB 400	None
C2	F, 28	ADNFLE	rMT, SRC, SICI, ICF, LICI	OXC 900, PER 10, PGB 225	None
C3	M, 50	Focal Cryptogenic	rMT, SRC, SICI, ICF, LICI	PHT 200, LCM 200	None
C4	F, 61	Focal Cryptogenic	rMT, SRC, SICI, ICF, LICI	PHT 400, LCM 600	None
C5	F, 47	Genetic Generalised	rMT, SRC, SICI, ICF, LICI	LTG 300, CLB 10 ZNS 200	None
C6	M, 44	Unclassified	rMT, SRC, SICI, ICF, LICI	LCM 400, OXC 1200 PER 2	None
C7	F, 27	Genetic Generalised	rMT, SRC, SICI, ICF, LICI	PHT 475, LMT 300	None
C8	M, 32	Genetic Generalised	rMT, SRC, SICI, ICF, LICI	VPA 2600, TPM 100, CLB 40	None
C9	M, 29	Focal Cryptogenic	rMT, SRC, SICI, ICF, LICI	LTG 100, CBZ 1200, PER 12, LEV 3000	None
C10	F, 21	Focal Cryptogenic	rMT, SRC, SICI, ICF, LICI	LEV 1500	None
**Healthy control subjects**					
Subject number	Sex, Age	Epilepsy Diagnosis	Testing Performed	Medication at time of testing (mg/day)	Intellectual Difficulties
C11	F,34	N/A	rMT, SRC, SICI, ICF, LICI	None	None
C12	F, 36	N/A	rMT, SRC, SICI, ICF, LICI	None	None
C13	M, 35	N/A	rMT, SRC, SICI, ICF, LICI	None	None
C14	F, 30	N/A	rMT, SRC, SICI, ICF, LICI	None	None
C15	M, 32	N/A	rMT, SRC, SICI, ICF, LICI	None	None
C16	M, 42	N/A	rMT, SRC, SICI, ICF, LICI	None	None
C17	M, 27	N/A	rMT, SRC, SICI, ICF, LICI	None	None
C18	F, 30	N/A	rMT, SRC, SICI, ICF, LICI	None	None
C19	M, 29	N/A	rMT, SRC, SICI, ICF, LICI	None	None
C20	M, 23	N/A	rMT, SRC, SICI, ICF, LICI	None	None

* indicates neuropsychometry assessment unavailable, intellectual difficulties assessed by clinician blinded to study outcome.

Abbreviations

rMT = motor threshold

SRC = stimulus-response curve

SICI = short interval intracortical inhibition

ICF = intracortical facilitation

LICI = long interval intracortical inhibition

TPM = topiramate

PHT = phenytoin

BAC = baclofen

FLN = flunarizine

PZT = pizotifen

ZNS = zonisamide

VPA = valproate

LTG = lamotrigine

PGB = pregabalin

AZM = acetazolamide

OMP = omeprazole

LTD = loratidine

OXC = oxcarbazepine

PER = perampanel

LCM = lacosamide

CLB = clobazam

LEV = levetiracetam

ADNFLE = autosomal dominant nocturnal frontal lobe epilepsy.

Table 1 shows their demographic information, genetic diagnosis (for those with AHC), epilepsy diagnosis (for epilepsy controls), medication at time of testing and degree of intellectual difficulties. There were no significant differences in age or proportions of males to females between the AHC group and either control group (age: unpaired, two-tailed t-test, P = 0.09 (AHC vs epilepsy controls), P = 0.07 (AHC vs healthy controls); sex: Fisher’s exact test P = 0.65 (AHC vs epilepsy controls), P = 0.37 (AHC vs healthy controls). Intellectual difficulties are common in AHC, and this is reflected in our population. Those with AHC were taking a mean of 2.1 medications each, while epilepsy controls were taking a mean of 2.5 medications each (see Table 1); there was no significant difference (unpaired, two-tailed t-test, P = 0.53).

### TMS during hemiplegic attacks

A1 was tested during a prolonged hemiplegic attack. Onset of left sided weakness occurred five days prior to testing, with partial resolution by the day of testing; mild ongoing weakness was evident on examination. Right-sided symptoms had occurred for two days prior to testing and had not resolved, with moderate weakness evident at the time of testing. His legs were mildly affected and he was able to walk. No motor response could be obtained at intensities of up to 80% maximum stimulator output on either side. Higher strengths were not tolerated and he declined further testing with TMS when well. rMT above 80% is rare in the general population; in a study of rMT in 141 healthy people, using the same model TMS machine (but a different coil), two people (1.4%) had rMT over 80% [27]. A1 was not taking any medication at all at the time of testing.

**Fig 1:**
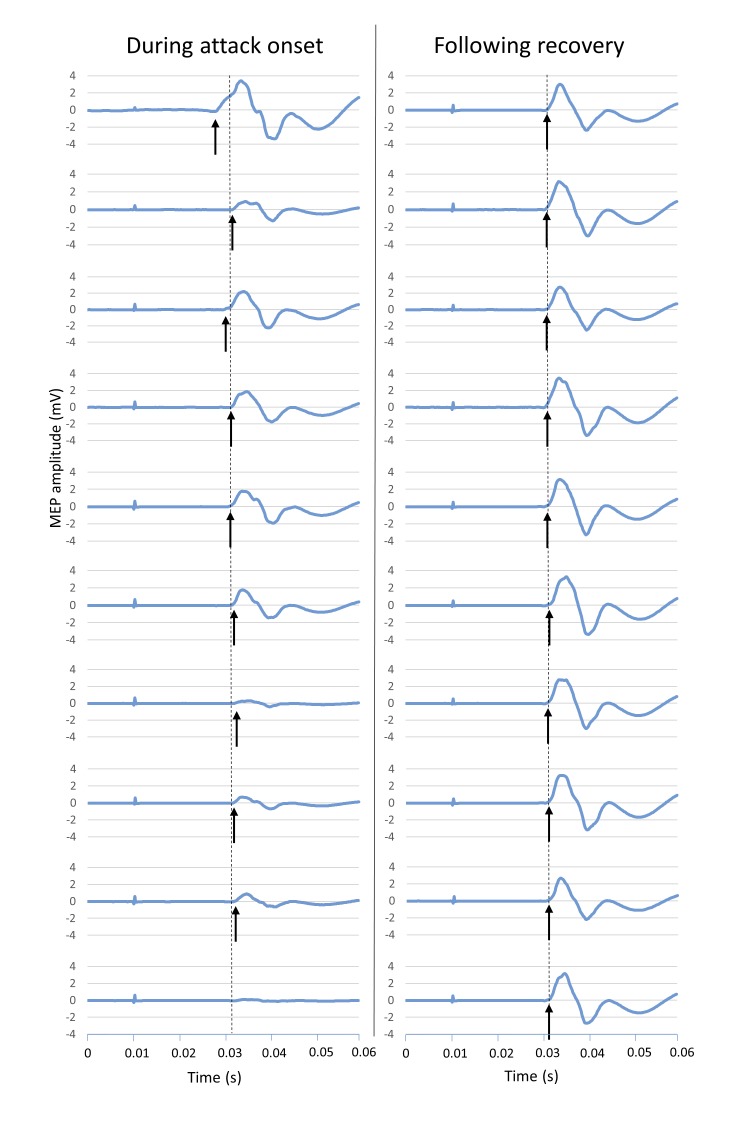
Motor Evoked Potentials during the onset of a hemiplegic attack. Consecutive MEPs during the onset of a hemiplegic attack (left panel) and following recovery, five minutes after the attack (right panel), in subject A2. Arrows represent latency of individual MEPs, dashed line represents average latency. During the onset of the attack (left panel), latency was variable, and amplitude decreased until no MEP was seen. Following recovery (right panel), amplitude and latency were stable. Stimulus strength was 130%rMT for all trials, which were performed at 5 second intervals.

A2 experienced an acute bilateral hemiplegic attack during her first TMS testing session. At the time, she was experiencing frequent (daily) brief bilateral attacks; her mother, who was present during testing, confirmed this attack was typical. During stimulation with an intensity of 130% rMT, motor responses gradually decreased over a one-minute period, until no response could be obtained. Testing was repeated five minutes later, following recovery, using the same stimulus intensity; no similar decrement in MEPs was found. These data are shown in Fig 1. MEP latency showed greater variability during the onset of hemiplegic symptoms compared to after recovery, although average latency was similar (see Fig 1).

### TMS between hemiplegic attacks

**Fig 2:**
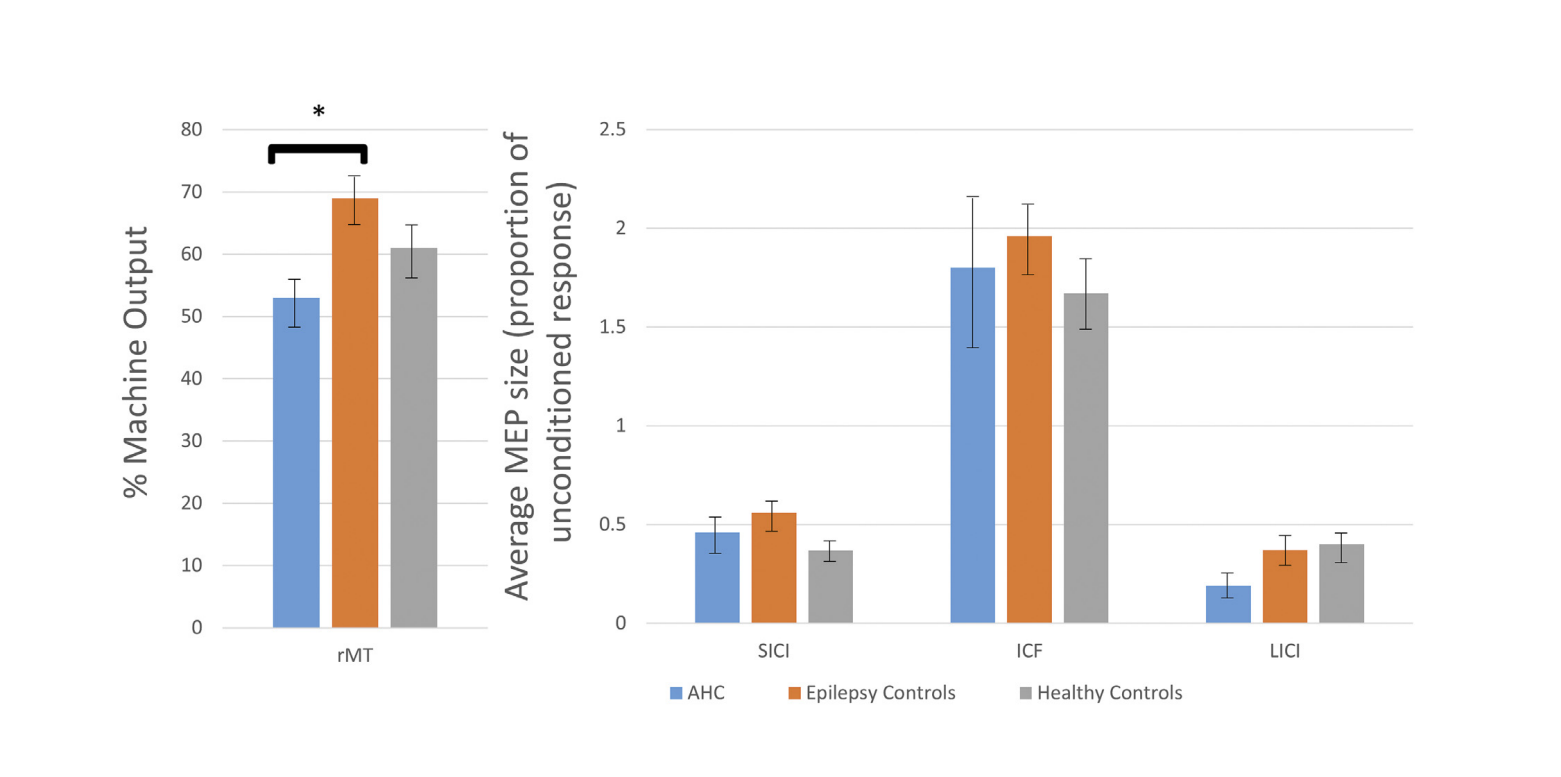
Mean results for resting motor threshold and paired pulse paradigms. Mean results for resting motor threshold (rMT), short interval intracortical inhibition (SICI), intracortical facilitation (ICF) and long interval intracortical inhibition (LICI) for people with alternating hemiplegia of childhood (AHC) and control subjects. There was a significant difference only in rMT (indicated by *, P = 0.002). Error bars show standard error.

rMT was measured in seven people with AHC without hemiplegic symptoms, or any other paroxysmal episodes, at the time of testing. Average rMT was 53% of maximum machine output, standard deviation (SD) 8.2%, range 38–64% (see Fig 2). This rMT is lower than the average rMT in our healthy control group (61%, range 37–82%), although the difference was not statistically significant (unpaired, two-tailed t-test P = 0.15). Controls with epilepsy had a significantly higher average rMT of 69% (SD 10.0%), (unpaired, two-tailed t-test P = 0.002), implying higher cortical excitability in the AHC group between hemiplegic attacks compared to people with epilepsy on treatment.

There was no significant difference between cases and controls in SICI, ICF or LICI, see Fig 2 (unpaired two-tailed t-tests: AHC vs epilepsy controls—SICI, P = 0.46; ICF, P = 0.70; LICI, P = 0.13; AHC vs healthy controls—SICI, P = 0.09; ICF, P = 0.75; LICI, P = 0.06).

**Fig 3:**
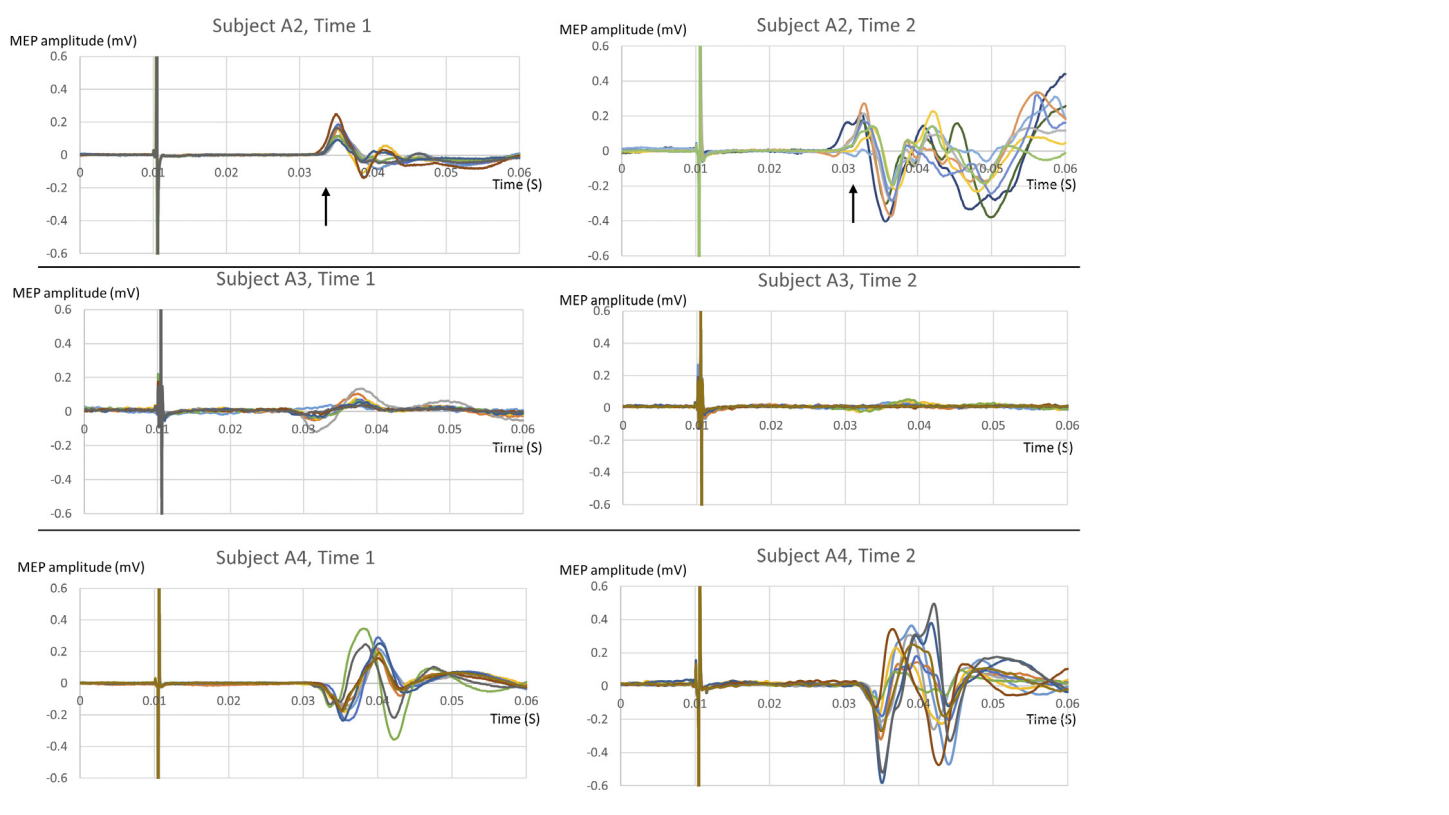
Variability in Motor Evoked Potentials in people with AHC. Each row shows two graphs of overlaid traces of 10 individual MEPs, performed using the same parameters, in the same subject, at different times during the same testing session. A change in MEP amplitude can be seen. In subject A2, there is also a change in MEP latency (average latency marked with an arrow).

Three out of five people with AHC who tolerated full testing (subjects A2, A3, A4) showed a discrete increase or decrease in MEP amplitudes at some point during a single testing session, without any hemiplegic symptoms, and without any change in stimulus size or other experimental conditions. Fig 3 compares ten traces recorded at the start of testing (labelled “Time 1”) with ten traces recorded after an interval (labelled “Time 2”), with a change in cortical excitability detected.

The change in amplitude was statistically significant in all three as shown in Table 2. Between Time 1 and Time 2, subjects were sitting relaxed in the testing room, undergoing TMS testing with other paradigms.

**Table 2:** Intra-individual variation in motor evoked potential amplitudes in people with AHC.

	Time 1		Time 2		
Subject	Mean Amplitude (mV)	SD	Mean Amplitude (mV)	SD	P value (2-tailed unpaired t-test)
A2	0.238	0.086	0.405	0.139	0.002
A3	0.137	0.065	0.053	0.024	0.008
A4	0.401	0.084	0.709	0.424	0.024

Mean amplitudes and standard deviation (SD) of MEPs at Time 1 and Time 2. Responses are to stimulation at 110% rMT. P values are for a two-tailed unpaired t-test comparing MEP sizes at Time 1 and Time 2. Note that in this small sample there is no consistent pattern such that rMT is always bigger at either Time 1 or Time 2.

Individual A2 also showed a statistically significant change in MEP latency from Time 1 to Time 2, shown in Fig 3 (Time 1, mean 22.97ms, SD 0.47ms; Time 2, mean 21.67ms, SD 1.02ms; two-tailed unpaired t-test, p<0.001). Subjects A3 and A4 did not show any change in latency. No change in excitability or latency was seen in two other participants with AHC who tolerated full testing, or in any controls.

Time 1 and Time 2 were less than 30 minutes apart. There was no accompanying clinical correlate, and none of these people experienced paroxysmal hemiplegic symptoms during testing; the bilateral hemiplegic attack in A2 occurred during an earlier testing session. A2 exhibited some dystonic posturing of the left arm between Time 1 and Time 2; recordings were taken from the left adductor pollicis brevis muscle. There was no significant change in pre-stimulus muscle activation between Time 1 and Time 2 (50ms pre-stimulus EMG quantified using a root mean squared model, compared using unpaired, 2-tailed t-tests). The site of stimulation was marked on a tight fitting latex cap that was not moved between testing sessions. In A3 and A4, the position of the coil was also confirmed using neuronavigation. There was no change in TMS stimulus intensity. All subjects were awake and responsive during testing, with their eyes open.

**Fig 4:**
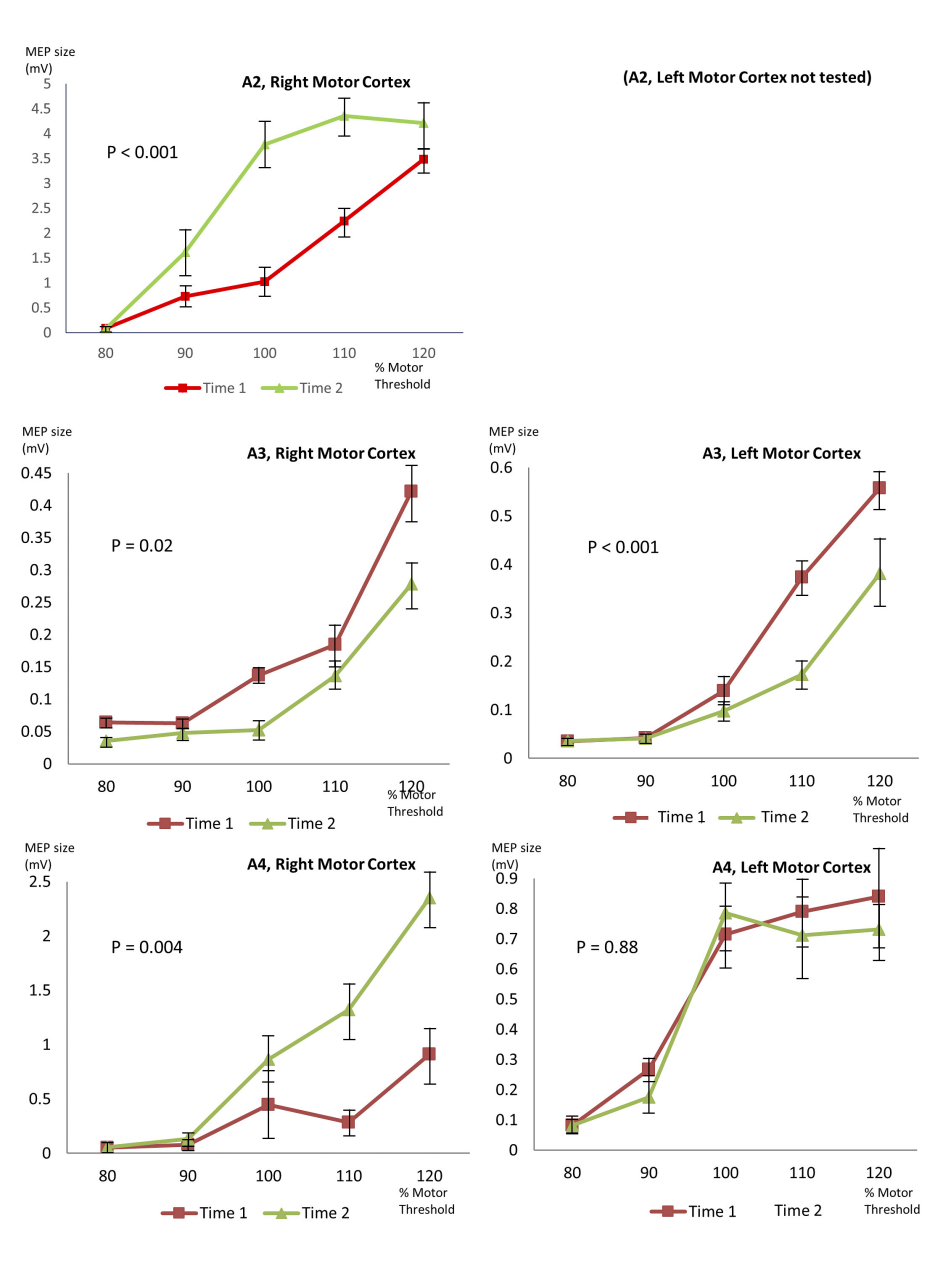
Variability in stimulus-response curves in people with AHC. Each graph shows two stimulus-response curves from the same subject, during the same testing session, demonstrating a change in cortical excitability. Subject 2 only tolerated testing of Right Motor Cortex, Subjects 3 and 4 were tested on both sides. P values given in the Fig were calculated using multivariate ANOVA comparing MEP size at Time 1 and Time 2, across stimulation intensities. Error bars show standard error.

Stimulus-response curves showed significant changes from Time 1 to Time 2 in A2 (only right hemisphere tested, P<0.001), A3 (right hemisphere P = 0.02, left hemisphere P<0.001) and A4 (right hemisphere P = 0.004, left hemisphere no significant change). The stimulus-response curves are illustrated in Fig 4. A3 and A4 had both hemispheres tested during the same testing session; in A3 a significant change in cortical excitability was evident in both hemispheres while in A4, a change was only seen in one hemisphere. Both increases and decreases in excitability were seen, in different participants.

**Fig 5:**
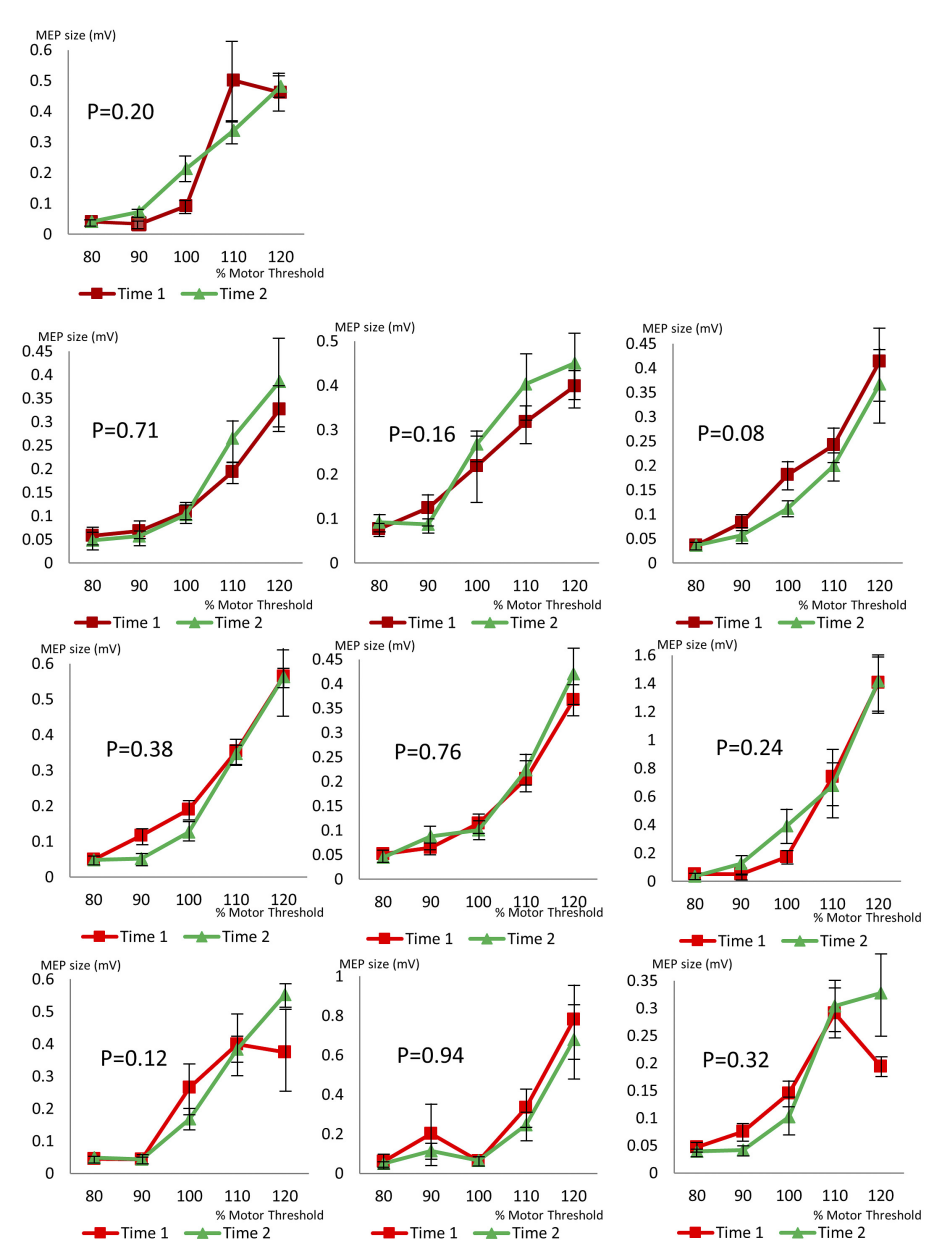
Stimulus-response curves for ten control subjects with epilepsy, but not AHC. Each graph shows two stimulus-response curves from the same subject, during the same testing session, demonstrating no significant change in cortical excitability. P values were calculated using multivariate ANOVA comparing MEP size at Time 1 and Time 2, across stimulation intensities. Error bars show standard error.

**Fig 6:**
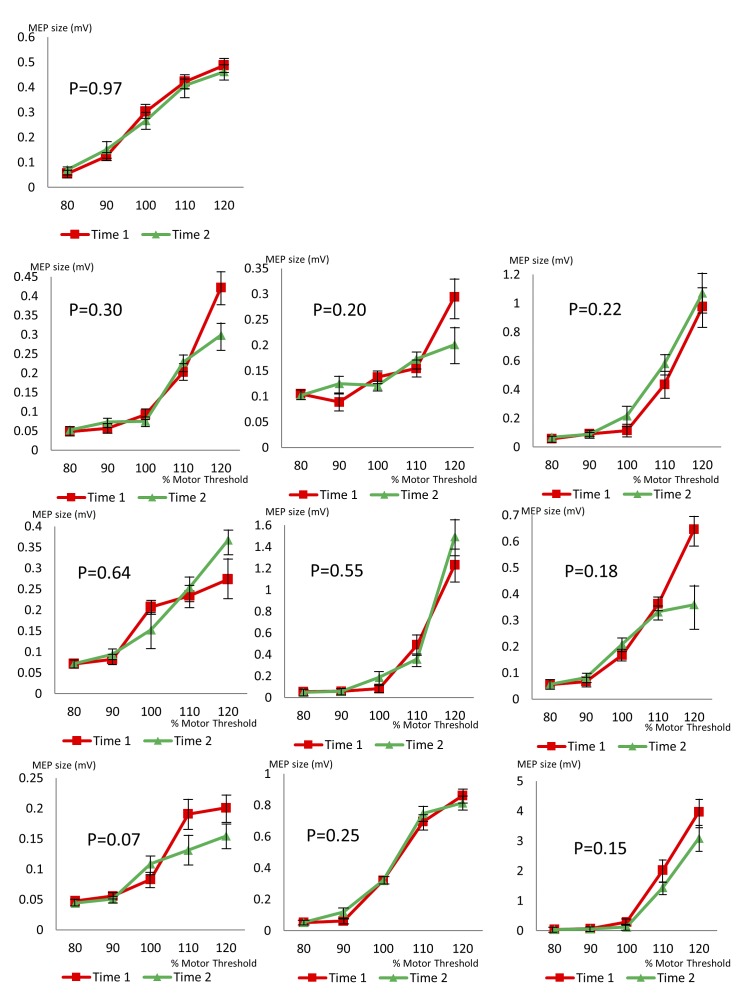
Stimulus-response curves for ten healthy control subjects. Each graph shows two stimulus-response curves from the same subject, during the same testing session, demonstrating no significant change in cortical excitability. P values were calculated using multivariate ANOVA comparing MEP size at Time 1 and Time 2, across stimulation intensities. Error bars show standard error.

Testing was performed in ten epilepsy controls and ten healthy controls using an identical experimental paradigm. None of them showed a significant change in cortical excitability during the testing period. Stimulus-response curves and P values for epilepsy controls are shown in Fig 5, and for healthy controls in Fig 6.

## Discussion

Our key finding is the fluctuation in cortical excitability seen in people with AHC, but not in controls. The fluctuation appears to affect the size and latency of MEP for a given stimulus strength and was seen in three of five people with AHC who tolerated full testing, but none of our 20 controls. This instability was most marked during the onset of a hemiplegic attack, but was also present without any obvious clinical correlate in asymptomatic individuals. No such fluctuation has been reported in TMS studies across several neurological conditions; for example, Parkinson’s disease [28], multiple sclerosis [29] and epilepsy [30]. The findings seem unique to AHC and may constitute an electrophysiological correlate of paretic or plegic attacks.

Two subjects were tested during a bilateral hemiplegic attack: no MEPs could be recorded in either. In A2, a brief bilateral hemiplegic attack occurred during testing, preceded and followed by a period of normal cortical excitability. A decline in cortical excitability during the onset of the attack was recorded, with consecutive MEPs falling in amplitude and fluctuating in latency until no MEP could be elicited, in parallel with the clinical development of a bilateral hemiplegic attack. Subject A2 was experiencing frequent brief bilateral hemiplegic attacks around the time of testing, and her mother (who was present) confirmed that the attack was typical. Her hemiplegic attacks do not affect alertness or level of consciousness.

A1 was tested only during a prolonged bilateral hemiplegic attack; he declined further testing when his symptoms had improved. During his attack, despite stimulation of both hemispheres up to the maximum tolerated (80% of total) stimulator output, no MEPs could be recorded. None of the other participants with AHC had an rMT above 60%. Taken together, these findings demonstrate low motor cortex excitability during hemiplegic episodes.

Three individuals with AHC showed variation in cortical excitability, without any simultaneous hemiplegic symptoms, as demonstrated by a change in MEP size and latency, and a shift in the stimulus-response curve. The stimulus-response curve is a commonly tested TMS parameter. Its stability in individuals is well documented, even when people are tested on different days [31,32]. Fluctuations of this nature during a single testing session have not been previously reported and we have not encountered similar results despite significant experience in the field.

Between attacks, people with AHC had rMT lower than our healthy volunteers, despite several of our participants with AHC taking medication known to increase rMT. This difference did not reach statistical significance, but is in the same direction as animal data suggesting increased cortical excitability in AHC [22]. Our disease controls, on a similar number of medications, had a significantly higher average rMT than the AHC group. We note, however, that medication in the two groups does not match exactly; more disease controls were taking sodium channel blockers, which are known to increase rMT, and this may account for the difference observed. Further investigation would require a larger dataset, and ideally would include TMS testing of people with AHC on no medication.

Results of paired pulse testing and cortical silent period were similar to controls, suggesting that intracortical excitatory and inhibitory function is relatively unaffected by AHC. Subtle abnormalities in these circuits might not be detected by our paradigms.

Some dynamic factors affect cortical excitability. Pre-activation of the target muscle increases excitability but this possibility was excluded by evaluation of the EMG traces. Mental imagery relating to movement of the target muscle can also reduce motor threshold [33], but no mental imagery of movement was encouraged. Participants might conceivably become increasingly relaxed as they acclimatised to testing. These factors should have applied equally to controls.

Accidentally changing position of the TMS coil during an experiment would affect the stimulus-response curve. An unintended change in position would move away from the motor hotspot, which should reduce MEP size. An increase (as seen in A2 and A4) could only be generated if the motor hotspot had originally been identified incorrectly, and the coil was then accidentally moved to a better stimulation site. The position of the coil was marked on a tight-fitting cap that was kept in place throughout testing. By comparison, many have reported TMS experiments in the same people performed on different days, which requires the motor hot-spot to be newly identified for each test. Few of these other reported experiments used neuronavigation and the same spot may not have been selected when the repeat experiments were performed in those studies. Nonetheless, those experiments achieved reliable data, and were sensitive enough to detect small changes in cortical excitability, such as those caused by a single dose of an anti-epileptic drug [34]. In two participants in whom we recorded a change in excitability, neuronavigation confirmed that there had been no change in position of the coil. Controls were tested under exactly the same conditions, and no similar change was seen. People with AHC and controls were all tested by experienced TMS operators (WMS, GS, DH, MD).

Of three participants in whom fluctuating excitability was recorded, two had proven mutations in *ATP1A3* (A2, A4). No mutation was found in A3 (tested previously with whole genome sequencing); around 20% of people with a clinical diagnosis of AHC have no detected mutation in *ATP1A3* [5]. An unknown second gene may lead to the same clinical and neurophysiological phenotype. Alternatively, some mutations affecting *ATP1A3* may be missed using current techniques.

Two people with AHC and known *ATP1A3* mutations, in whom full testing was possible, did not show fluctuating cortical excitability. Both these participants live independently and are at the mild end of the AHC spectrum. However, more data would be required to establish any possible link between neurophysiology and phenotype or mutation type in humans. An effect of *ATP1A3* mutation type on physiology has recently been demonstrated in Xenopus oocytes [20].

Our work has limitations. Inevitably in such a rare condition numbers are small. Only two subjects could be tested during hemiplegic episodes. Among those with AHC, not all were able to tolerate full testing. Severe intellectual difficulties may limit participation in TMS research and the subject we recruited with severe intellectual difficulties (A8) could not be tested; excluding such people may introduce selection bias. Several further individuals with AHC had intellectual and behavioural difficulties; some found TMS uncomfortable and did not complete testing. Those with mild intellectual difficulties who tolerated testing cooperated well. Fluctuating cortical excitability was seen in those with and without intellectual difficulties.

Some participants were taking neurologically-active medication (see Table 1). Our epilepsy controls were also taking medication; their MEPs and stimulus-response curves do not show similar variability (see Fig 5). Inevitably, the drugs taken by the AHC group and controls do not match exactly; for instance, flunarizine is used in AHC but not used in epilepsy. One participant in whom we measured a fluctuation was only taking baclofen, a drug known not to affect MT or MEP size [34]. No similar fluctuations during a single TMS testing session have been reported in extensive TMS data from healthy subjects given anti-epileptic and other drugs [34]. Participant A1 was on no medication when tested during a hemiplegic attack; medication effects cannot explain his low cortical excitability, and neither can they explain the transiently reduced cortical excitability seen in A2 during a hemiplegic episode.

rMT and the stimulus-response curve are complex and compound measures that rely on the entire motor pathway to be functioning. There is evidence that hemiplegic attacks in AHC have a central mechanism, but changes at any point from the motor cortex down to the muscles themselves could affect the recordings. ATP1A3 is expressed in the cortex but also in other brain regions, the spinal cord, motor nerves and muscle [35,36]. A recent study of somatosensory evoked potentials in people with AHC suggested possible disinhibition in the somatosensory system [4]. This phenomenon also appeared to show dynamic fluctuations, particularly during hemiplegic attacks. A recent knock-in mouse model with clinical features closely mimicking AHC showed both increased neuronal excitability (possibly predisposing to seizures) and increased predisposition to cortical spreading depression, which was suggested as a possible mechanism for hemiplegic attacks [22]. We recently showed abnormalities in the ECG in AHC, with characteristics suggesting defective repolarisation reserve: the abnormalities were dynamic, increasing in prevalence with age, but also changing over time, and even beat-to-beat, in the same individual [3]. We propose that AHC is characterised by sub-clinical dynamic instability in excitable tissues, which can become clinically manifest as hemiplegic episodes and occasionally dysfunction in other affected tissues. This hypothesis is testable in some existing models, and further work will be needed to explore how these dynamic fluctuations are caused by the mutant ATP1A3 protein.

## Conclusions

This study used TMS to study motor cortex excitability in people with AHC. Low excitability was found during hemiplegic attacks. Between attacks, cortical excitability showed asymptomatic fluctuations, not seen in controls. We hypothesize that the asymptomatic excitability changes we observed were due to altered function of the ATP1A3 mutant protein, and that larger fluctuations, either spontaneous or triggered by external factors, cause hemiplegic attacks. It is possible that the observed fluctuation might act as a biomarker for disease activity, providing information as a surrogate for hemiplegic attacks that occur on a much longer time scale. Such a biomarker might be of value, for example, for trials of novel treatments for AHC.

## Supporting Information

S1 Table Results for rMT (motor threshold), SICI (short interval intracortical inhibition), ICF (intracortical facilitation), LICI (long interval intracortical inhibition) for each participant. (DOCX)

## Abbreviations

AHC: alternating hemiplegia of childhood
SPECT: single-photon emission computed tomography
TMS: transcranial magnetic stimulation
MEP: motor evoked potential
APB: adductor pollicis brevis
rMT: resting motor threshold
SICI: short interval intracortical inhibition
ICF: intracortical faciliation
LICI: long interval intracortical inhibition
SD: standard deviation
