# Supplementary material for: Spontaneously Fluctuating Motor Cortex Excitability in Alternating Hemiplegia of Childhood: A Transcranial Magnetic Stimulation Study
Source: PLoS One. 2016 Mar 21;11(3):e0151667. doi: 10.1371/journal.pone.0151667 (PMC4801356; doi:10.1371/journal.pone.0151667)
Supplement: S1 Table — (DOCX) [file pone.0151667.s001.docx]

| Number | rMT  (% machine output) | SICI | ICF | LICI |
| --- | --- | --- | --- | --- |
| A1 | >80 during attack | NT | NT | NT |
| A2 | 56 | 0.5 | 0.71 | 0.12 |
| A3 | 58 | 0.6 | 1.71 | 0.31 |
| A4 | 52 | 0.55 | 2.27 | 0.12 |
| A5 | 52 | NT | NT | NT |
| A6 | 64 | 0.34 | 3.31 | 0.19 |
| A7 | 38 | NT | NT | NT |
| A8 | NT | NT | NT | NT |
| A9 | 48 | 0.33 | 1 | 0.22 |
| **Average (Standard Deviation)** | **53 (8.22)** | **0.46 (0.12)** | **1.80 (1.04)** | **0.19 (0.08)** |
| C1 | 64 | 0.52 | 1.57 | 0.45 |
| C2 | 85 | 0.87 | 1.90 | 0.14 |
| C3 | 70 | 0.67 | 1.78 | 0.83 |
| C4 | 85 | 0.72 | 3.02 | 0.11 |
| C5 | 56 | 0.30 | 1.20 | 0.20 |
| C6 | 62 | 0.85 | 1.96 | 0.59 |
| C7 | 62 | 0.18 | 2.24 | 0.39 |
| C8 | 65 | 0.73 | 1.72 | 0.51 |
| C9 | 78 | 0.44 | 2.89 | 0.20 |
| C10 | 66 | 0.34 | 1.38 | 0.26 |
| **Average (Standard Deviation)** | **69 (10.06)** | **0.56 (0.24)** | **1.96 (0.60)** | **0.37 (0.23)** |
| C11 | 63 | 0.22 | 1.45 | 0.82 |
| C12 | 65 | 0.54 | 2.86 | 0.21 |
| C13 | 82 | 0.38 | 1.07 | 0.45 |
| C14 | 57 | 0.43 | 0.33 | 0.25 |
| C15 | 76 | 0.42 | 1.77 | 0.09 |
| C16 | 37 | 0.36 | 1.59 | 0.38 |
| C17 | 56 | 0.23 | 1.48 | 0.75 |
| C18 | 63 | 0.40 | 2.46 | 0.47 |
| C19 | 49 | 0.42 | 2.16 | 0.19 |
| C20 | 62 | 0.28 | 1.38 | 0.36 |
| **Average (Standard Deviation)** | **61 (12.70)** | **0.36 (0.10)** | **1.66 (0.72)** | **0.40 (0.24)** |

Supporting Table 1: Results for rMT (motor threshold), SICI (short interval intracortical inhibition), ICF (intracortical facilitation), LICI (long interval intracortical inhibition) for each participant. SICI, ICF and LICI are expressed as a ratio of MEP size with conditioning stimulus compared to stimulus alone, with values <1 implying inhibition and values >1 implying facilitation. NT = not tested. Averages for each group are given with standard deviation in brackets.
